# Supplementary material for: Engineered Extracellular Vesicles with Compound-Induced Cargo Delivery to Solid Tumors
Source: Int J Mol Sci. 2023 May 27;24(11):9368. doi: 10.3390/ijms24119368 (PMC10253715; doi:10.3390/ijms24119368)
Supplement: Supplementary file 1 [file ijms-24-09368-s001.zip › ijms-2326216-supplementary.pdf]

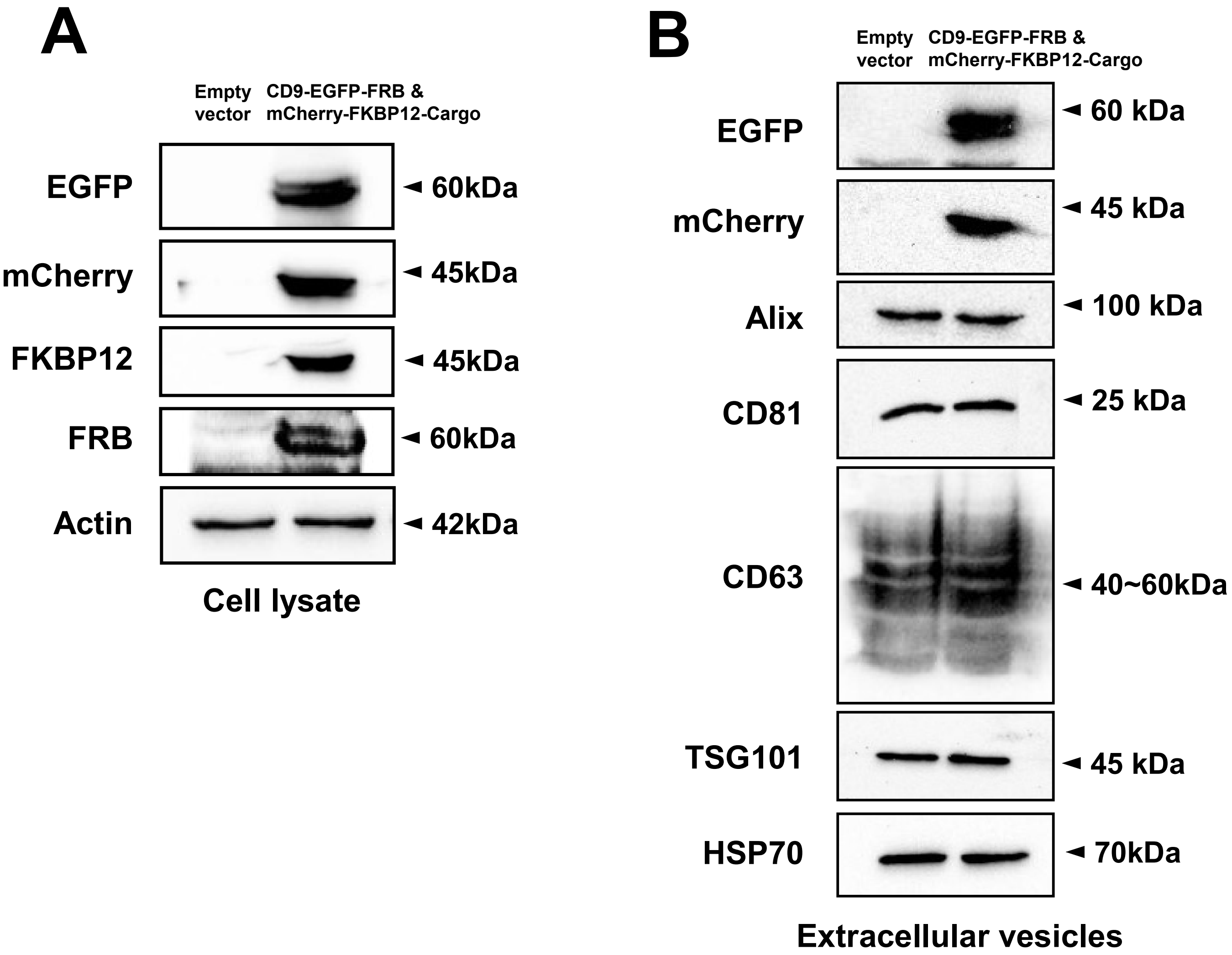

Supplemental Figure S1. The expression of vectors encoding CD9-EGFP-FRB and mCherry-FKBP12-Cargo in COS-7 cells

The COS-7 cells were transiently transfected with expression vectors encoding both CD9-EGFP-FRB and mCherry-FKBP12-Cargo. (A) COS-7 cell lysates were separated by SDS-PAGE and then analyzed with indicated antibodies such as EGFP, mCherry, FKBP12, FRB and Actin. Actin was used as a loading control. (B) The released EVs obtained from rapamycin-treated COS-7 cells were separated by SDS-PAGE and then analyzed with indicated antibodies such as EGFP, mCherry, Alix, HSP70, CD81, CD63 and TSG101. Alix, HSP70, CD81, CD63, and TSG101 were enriched in EVs and then used as their markers.

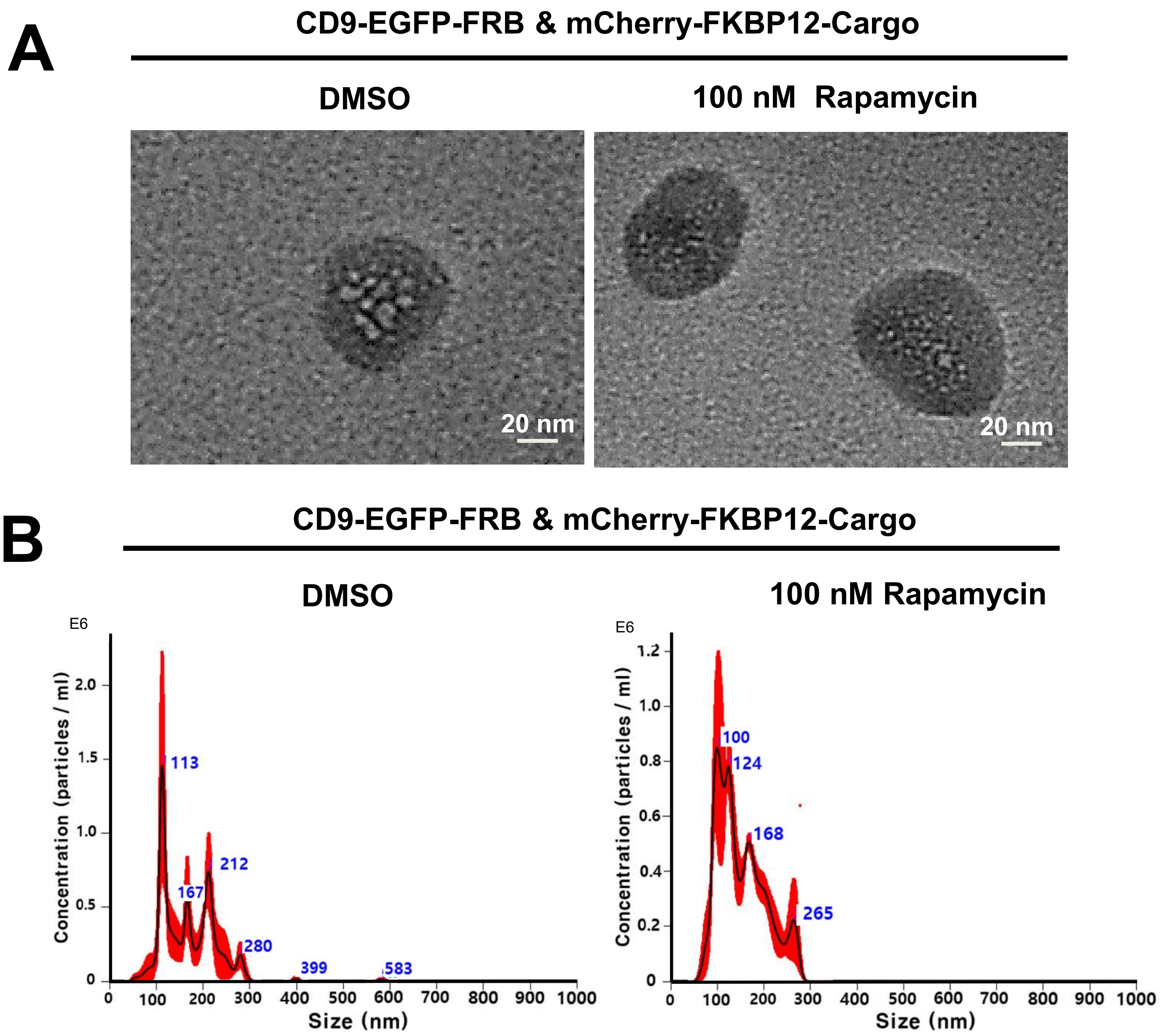

Supplementary Figure S2. Characteristics of engineered extracellular vesicles isolated from COS-7 cells transfected with CD9-EGFP-FRB and mCherry-FKBP12-Cargo.

(A) The images of transmission electron microscopy (TEM) of the released EVs obtained from COS-7 cells transfected with two plasmids encoding CD9-EGFP-FRB and mCherry-FKBP12-Cargo. The images were shown in the absence or presence of 100 nM Rapamycin. The scale bar is 20 nm. (**B)** The distribution of EVs was measured in the absence or presence of 100 nM rapamycin. The black graph indicates an average finite track length adjustment (FTLA) concentration/size. Red color shows the standard errors of mean.

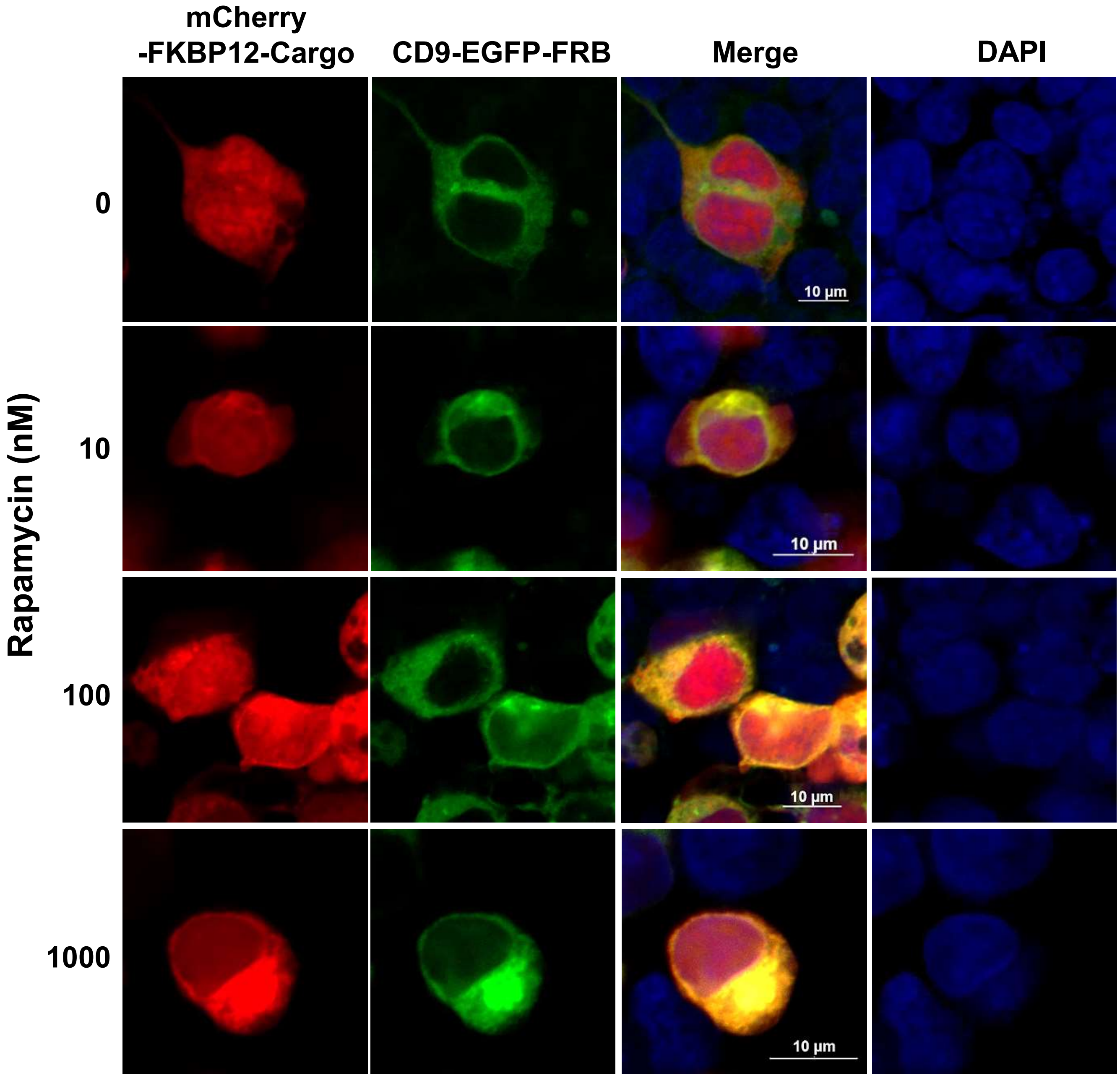

Supplementary Figure S3. The co-localization of mCherry-FKBP12-Cargo and CD9-EGFP-FRB in rapamycin-dose dependent manner in HEK-293 cells.

The HEK-293 cell were transfected with the expression vectors encoding with CD9-EGFP-FRB and mCherry-FKBP12. These cells were incubated with the indicated concentration of rapamycin for 24 h. The subcellular co-localization was captured with confocal microscopy. Scale bar, 10 µm.

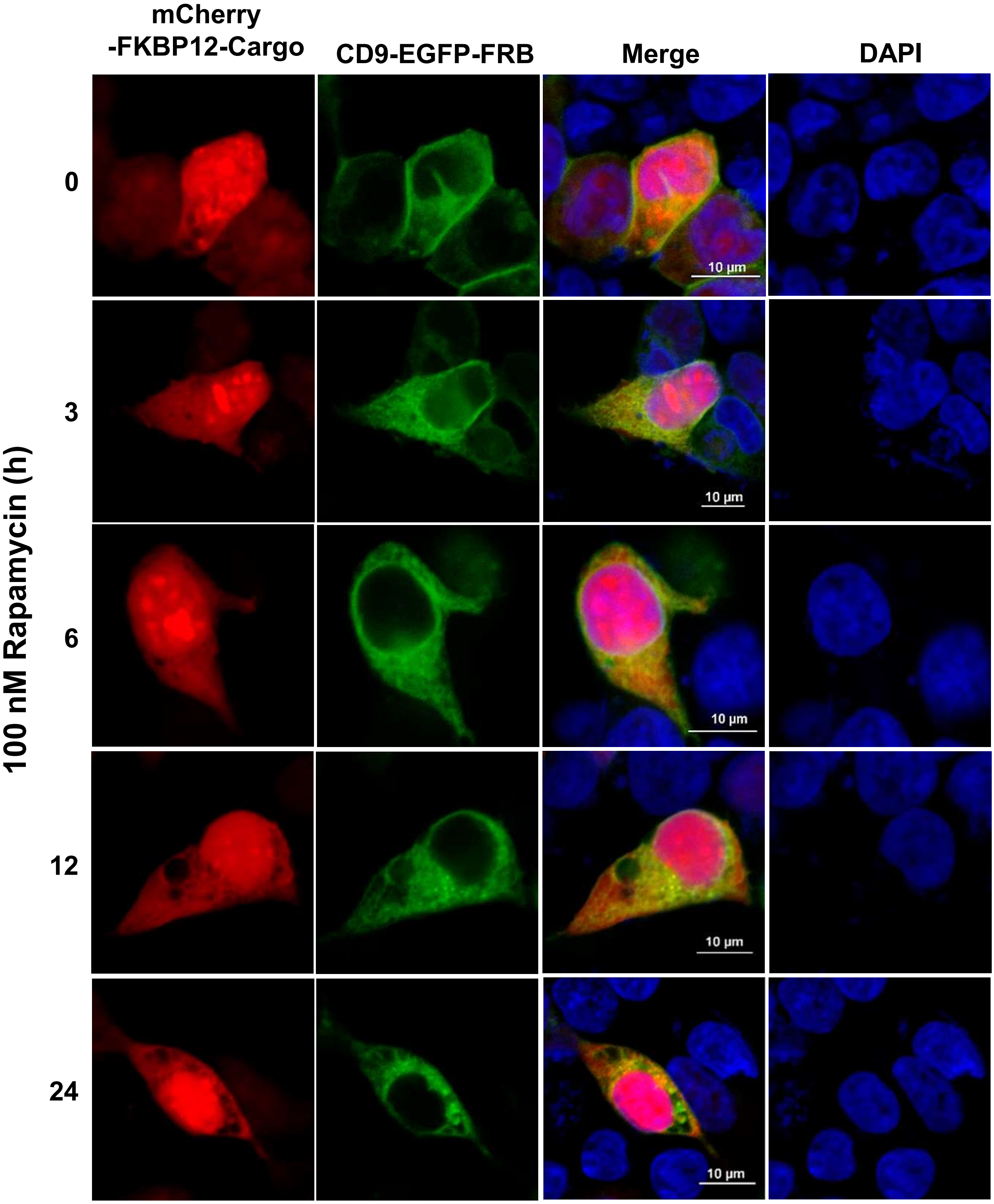

Supplementary Figure S4. The co-localization of mCherry-FKBP12-Cargo and CD9-EGFP-FRB according to treatment time of rapamycin in HEK-293 cells.

The HEK-293 cell were transfected with the expression vectors encoding with CD9-EGFP-FRB and mCherry-FKBP12. These cells were incubated with the indicated time treated with rapamycin (100 nM). The subcellular co-localization was captured with confocal microscopy. Scale bar, 10 μm.

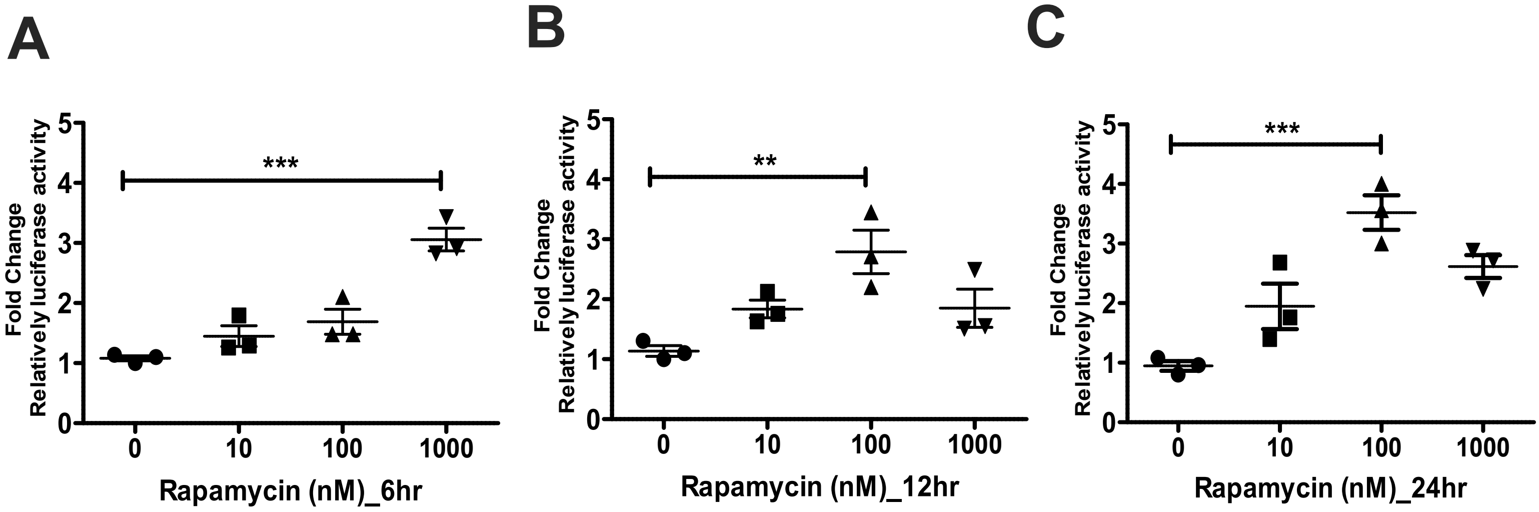

Supplementary Figure S5. The luciferase activity

HEK-293 cells were transfected with the expression vectors encoding mCherry-FKBP12-luciferase and CD9-EGFP-FRB. The released EVs were isolated in cells treated with the indicated concentration and time of rapamycin and then the luciferase activity was measured. (A) was treated for 6 h, (B) was treated for 12 h, and (C) was treated for 24 h. Data were analyzed by one-way ANOVA, followed by Turkey's HSD test. mean ± s. e. (N = 3). *** $p = 0.0002$ for 6 h, **$p < 0.0125$ for 12 h, and ***$p = 0.0007$ for 24 h)

Figure 2A

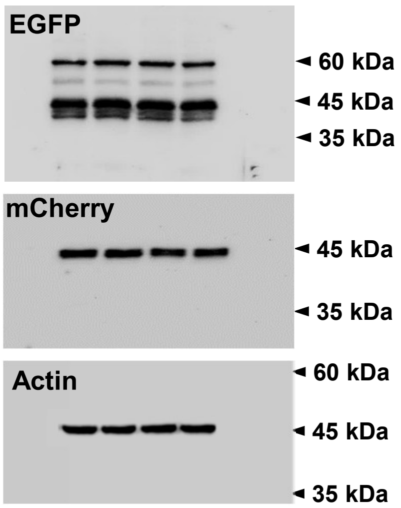

Figure 2C

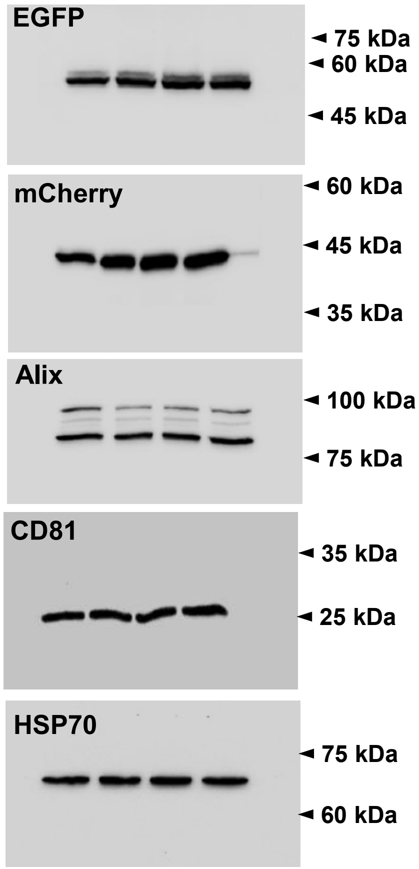

Figure 2E

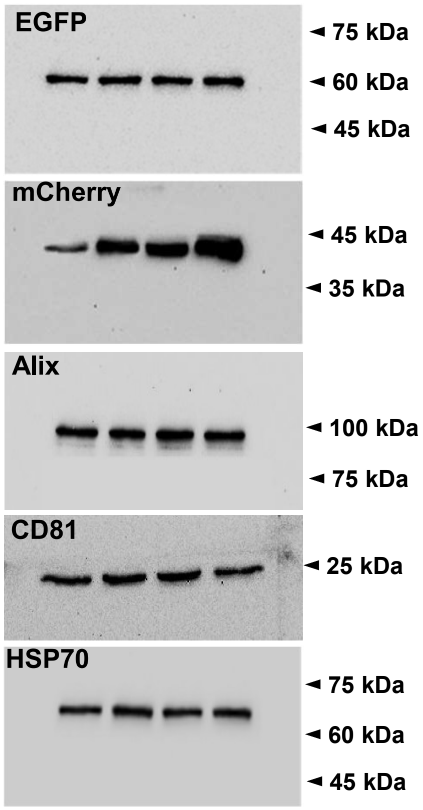

Supple. Figure 1A

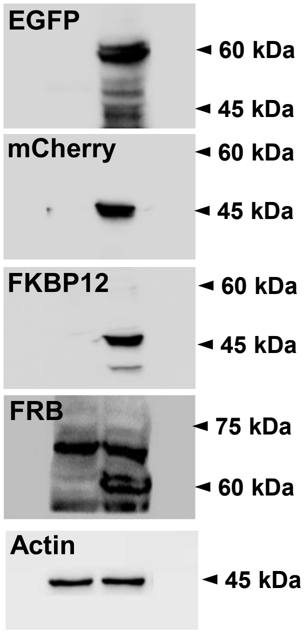

Supple. Figure 1B

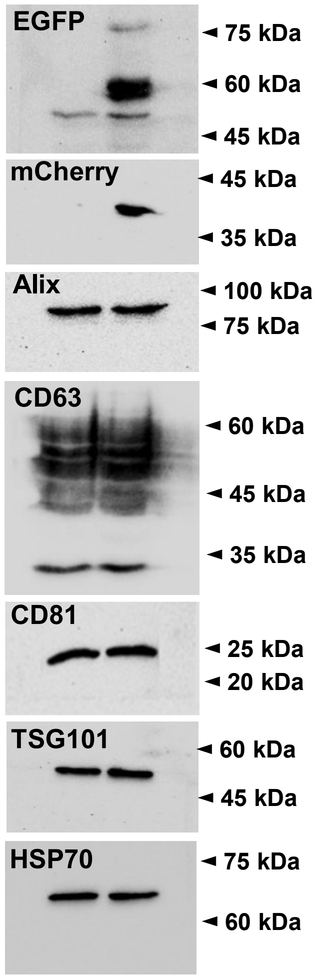

Supplementary Figure S6. Raw data of immunoblots used in this study
